# Changes in EEG Alpha Activity during Attention Control in Patients: Association with Sleep Disorders

**DOI:** 10.3390/jpm11070601

**Published:** 2021-06-25

**Authors:** Anastasiya Runnova, Anton Selskii, Anton Kiselev, Rail Shamionov, Ruzanna Parsamyan, Maksim Zhuravlev

**Affiliations:** 1 Department of Basic Research in Neurocardiology, Institute of Cardiological Research, Saratov State Medical University Named after V.I. Razumovsky, B. Kazachaya Str., 112, 410012 Saratov, Russia; runnova.ae@staff.sgmu.ru (A.R.); selskiiao@gmail.com (A.S.); antonkis@list.ru (A.K.); kvl.PRR@mail.ru (R.P.); 2 Institute of Physics, Saratov State University, Astrakhanskaya Str., 83, 410012 Saratov, Russia; 3 National Medical Research Center for Therapy and Preventive Medicine, 10, Petroverigsky per., 101953 Moscow, Russia; 4 Faculty of Psychological, Pedagogical and Special Education, Saratov State University, Astrakhanskaya Str., 83, 410012 Saratov, Russia; shamionov@mail.ru

**Keywords:** EEG, wavelet-analyse, alpha-activity, attention, sleep disorder, insomnia

## Abstract

We aimed to assess which quantitative EEG changes during daytime testing in patients with sleep disorder (primary insomnia and excessive daytime sleepiness groups). All experimental study participants were subjected to a long-term test for maintaining attention to sound stimuli, and their EEGs were recorded and then processed, using wavelet analysis, in order to estimate the power and frequency structure of alpha activity. In healthy subjects, the maximum increase in the alpha rhythm occurred near 9 Hz. Patients with primary insomnia were characterized by an increase in the amplitude of the alpha rhythm near 11 Hz. For subjects with sleep disorders, an increase in the amplitude of the alpha rhythm was observed in the entire frequency range (7.5–12.5 Hz), with a maximum increase at 9–10 Hz. Significant differences (p≤0.001) for changes in the alpha rhythm dynamics in the course of performing the attention test were observed in the frequency range of 7.5–10.5 Hz between the control group and patients with sleep disorders. The ratios of the alpha rhythm power values for passive stages with closed eyes before and after active stage were significantly different among the groups of healthy sleep volunteers, patients with primary insomnia, and patients with impaired sleep hygiene within the range of 9.5 to 12.5 Hz. The results of the current study supported the notion of a 24-h hyperarousal in primary insomnia.

## 1. Introduction

Although the prevalence of chronic primary insomnia in the general population is approximately 10%, transient symptoms of sleep disorders are detected much more often—in 30–35% of people [1,2]. Despite the effectiveness of contemporary psychotherapeutic and drug treatment contemporary psychotherapeutic and medicamentous treatment methods on many occasions, they are all associated with significant limitations [3,4,5,6,7,8]. A better understanding of the primary insomnia pathophysiology appears useful in assessing the potential of various treatment techniques, and also in developing novel approaches that specifically target neurophysiological characteristics correlating with primary insomnia symptoms. To date, the change in neurophysiological characteristics of patients with insomnia in the presleep period and during their sleep has been sufficiently studied [9,10,11,12,13]. Physiological markers may include an increase in high EEG frequencies in the peri-sleep onset period, as well as during non-rapid eye movement sleep and rapid eye movement phase [10,12,14], along with reduction in the slow-wave sleep [15,16] and power spectra decrease in delta and theta bands in some subjects [10,13,17].

At the same time, modern somnology suggests that primary insomnia is more a condition of 24-h hyperarousal than a purely nocturnal disorder [18]. In particular, it has been shown that patients with insomnia, in spite of their subjective complaints for daytime somnolence, as well as their significantly shorter nocturnal sleep, do not exhibit augmented somnolence, as compared with normal sleepers [19,20,21]. Recent evidence suggests that patients with primary insomnia show changes in neurophysiological activity during normal daytime wakefulness compared to those with normal sleep [22,23,24]. However, a very high individual variability of the brain electrical activity significantly reduces the ability to identify such traits across patients [25]. Currently, lower values of theta power and higher values of beta power in patients with primary insomnia were established [24]. However, the presented characteristics of EEG activity are weakly specific, since they can be observed in normal sleepers as well, for example, in conditions of visual fatigue accumulation [26,27] or with major depressive disorder [28].

In this study, we present a quantitative description of the change in the alpha rhythm (as electroencephalography biomarker) during the daytime in patients with primary insomnia. Despite the fact that the EEG activity parameters are extremely variable across the population [29,30], it is the alpha rhythm that is a very stable trait in subjects of different ages [31,32]. It is well known that the alpha rhythm activity increases with a decrease in the levels of sensory signals: e.g., when eyes are closed, the alpha rhythm power increases in the occipital brain lobe [33]. At the same time, it is known that the alpha rhythm amplitude changes in the course of cognitive activities, such as cognitive performance, visual attention, memory, and complex abstract tasks [26,34,35,36,37,38]. Besides, it has been shown that the alpha rhythm during sleep undergoes significant changes in patients with insomnia [39,40].

Hence, the main goal of our study was to identify objective quantitative changes in the electroencephalography occurring during the daytime activity of patients suffering from insomnia. For the first time, the comparison of patients with primary insomnia is conducted not only with a control group of apparently healthy subjects, but also with a group of patients suffering from insomnia caused by poor sleep hygiene.

## 2. Materials and Methods

### 2.1. Data and Materials

The participants volunteered in our experiments on a complimentary basis. All study subjects signed an informed medical consent to participate in the experimental work, received all necessary explanations about the research, and agreed to the subsequent publication of the study results. Collected experimental data were processed sensu the principles of confidentiality and anonymity of research participants. The design of experimental studies was approved by the local scientific research Ethics Committee. The experimental study subjects were recruited from the outpatients with sleep disorders at the Pain Management Clinic (Saratov, Russian Federation). The inclusion criteria for our study were as follows:

- Male gender, age <25 years;
- Complaints for insufficient and non-restorative sleep;
- Sleep onset disorder (>30 min to fall asleep) or sleep maintenance disorder (two or more awakenings per night of >15 min long or wake after sleep onset (WASO) time of >30 min);
- Problem incidence rate >3 nights per week;
- Problem duration >6 months.

Exclusion criteria were as follows:

- Beck Depression Inventory score (BDI) >13 [41];
- A score >7 on the Hospital Anxiety and Depression Scale [42];
- An apnea-hypopnea index (AHI) or periodic limb movements index (PLM) >5 or restless leg syndrome (RLS) during the polysomnography night;
- A medical or psychiatric disorder;
- Psychotropic medicine use over the last month.

A sleep diary was kept daily for a week by every participant. All patients underwent neuropsychological status assessment by means of the Schulte Table test, Digital Symbol Substitution Test (DSST), Hospital Anxiety and Depression Scale (HADS), somnolence and sleep quality test; as well as tests for semantic and phonemic awareness, and memory. A comparative assessment of the cognitive functions in study participants via DSST, did not reveal a significant difference in indicators; all patients successfully completed the task with no more than two errors in 90 s, which was acceptable. It was also noted that all participants were successful in their studies at various universities (Saratov, Russia). The sleep interviews and the medical examination were conducted by the physicians, certified in neurology and sleep medicine, whereas the psychiatric interviews were conducted by board certified psychiatrists. Chronic insomnia disorder was diagnosed sensu 2014 International Classification of Sleep Disorders-Third Edition.

The study included three groups of subjects. The control group comprised of 15 patients with healthy sleep (Group I, n=15; age: 22.5±1.5 years; BMI: 20.3±1.2 kg/m${}^{2}$; BDI: 6±3; HADS: 5±1.2). The primary insomnia group included 12 men with chronic insomnia (Group II, n=12; age: 22.6±1.4 years; BMI: 20±1.5 kg/m${}^{2}$; BDI: 7±3; HADS: 6±0.5). Test subjects in Group II complained of pre- and intrasomnic disorders. At the same time, according to their sleep diaries, they tried to provide themselves with adequate conditions for normal sleep. The daytime somnolence group consisted of 5 patients with chronic insomnia and regular impaired sleep hygiene (Group III, n=5; age: 22.8±1.2 years; BMI: 20±1.1 kg/m${}^{2}$; BDI: 7±1; HADS: 5.2±0.8). The main complaint in Group III was daytime sleepiness. An average age of the subjects was 21 years 3 months. The ailment duration averaged 1.5 years.

Analyzing anamneses, we discovered that 87% of patients in Groups II and III sleep less than 5–6 h a day. In Group II, 97% of patients experienced difficulty falling asleep; 85% of subjects were spending over an hour before falling asleep; shallow sleep with frequent nocturnal awakenings was observed in 73% of patients; and 86% of patients were dissatisfied with the quality of their sleep. In Group III, 100% of patients had unbearable somnolence during daytime hours, causing difficulties in performing their duties.

The experiments were carried out in the late afternoon hours at a specially equipped laboratory. The day before and on the day of the experiment, every test subject did not have any episodes of a daytime sleep. During the experiment, while the participants were lying comfortably in a relaxed mode, their brain activity was measured (via EEG) in three different states: (1) stage of passive wakefulness with closed eyes (CY, duration of about 6–7 min), (2) passive wakefulness with periodic sound stimuli (*A*, around 35 min), (3) repeated stage of passive wakefulness with closed eyes (RCY, approximately 6–7 min). During the A-stage, all test subjects were instructed to stay awake predominantly with eyes open. In order to estimate their state of awakening and sleep, they were instructed to press a specific button on the remote control after each sound signal. Beeping sound signals alternated with pauses, the duration of which was set randomly in the range 7 to 14 s. We automatically estimated the duration of time interval $T_{r}$ between the sound signal and the test subject response (i.e., pressing the button). Time recordings for sound signals, pressing the button, and EEG data were conducted simultaneously.

**Figure 1:**
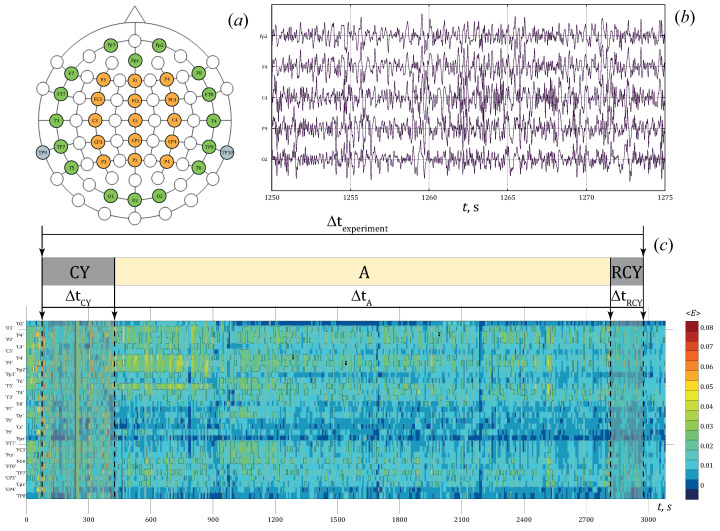
(**a**) The scheme of the “10–10” EEG electrode arrangement; (**b**) Fragments of EEG signals recorded during the experimental active stage; (**c**) The time dependence of the wavelet-energy in the band [8;12] Hz by EEG.

The multichannel surface EEG data were collected using the Encephalan-EEGR-19/26 recorder (Medicom MTD Ltd, Russia). Data were recorded at 250 Hz sampling rate using the conventional monopolar method of registration with two referential points and N=31 electrodes, as shown in Figure 1a. The adhesive Ag/AgCl electrodes in prewired head caps were used to obtain the EEG signals. Two reference electrodes, A1 and A2, were located on mastoids, while the ground electrode N was placed above the forehead. The EEG signals were filtered by a band-pass filter with cut-off points at 0.5 Hz (HP) and 70 Hz (LP) and a 50 Hz notch filter. Parts of EEG signals are presented in Figure 1b.

### 2.2. Methods

EEG recordings were processed by using the method of spatio-temporal analysis of multichannel EEG signals, based upon continuous wavelet transform (CWT) [43,44]. Currently, CWT is considered one of the most effective tools for studying the signals of biological objects [44,45]. We denote CWT in the following form:

(1) $$W\left(f,t_{0}\right)=\frac{1}{\sqrt{s}}\int_{-\infty }^{\infty }x\left(t\right)\psi_{f,t_{0}}^{*}\left(\frac{t-t_{0}}{f}\right)dt;$$

where x(t) is the experimental signal, $\psi_{f,t_{0}}^{*}\left(t\right)$ is the wavelet base function, *f* is the analog of ordinary frequency in Fourier analysis, symbol “*” denotes the complex conjugation. We use the Morlet wavelet as the CWT base function:

(2) $$\psi_{0}\left(\eta \right)=\frac{1}{\sqrt[{4}]{\pi }}e^{j2\pi \eta }e^{-\frac{\eta^{2}}{2}};$$

Morlet wavelet is widely used for analyzing the signals of biological objects, allowing to maintain the optimal ratio between the frequency and time resolution achieved with CWT (1). Each one-dimensional signal x(t) allows estimating a two-dimensional wavelet surface as:

(3) $$W\left(f,t_{0}\right)=\left|W(f,t_{0})\right|e^{j\phi f(t_{0})};$$

This surface $W(f,t_{0})$ characterizes the oscillatory activity for each frequency *f* at any time $t_{0}$ for the initial signal x(t). Next, we define the integrated energy distribution $\langle E(t_{0})\rangle$ for a certain frequency range $\left[f_{min};f_{max}\right]$ as:

(4) $$\langle E(t_{0})\rangle =\int_{f_{min}}^{f_{max}}{\left|W(f,t_{0})\right|}^{2}df;$$

We consider the frequency range of the classic alpha rhythm, describing oscillatory activity in frequency range $\Delta f_{\alpha }$, f∈(8;12) Hz [45,46]. In the context of this study, we divided this frequency range into 1 Hz bands.

The function $\langle E(t_{0})\rangle$ (4) is calculated for each of the EEG signal. To reduce the amount of data and the required time for numerical calculation, we averaged this function in a time window Δt = 5 s as

(5) $$\langle E_{k}\left(n,t_{0}^{\prime }\right)\rangle =\int_{t_{0}}^{t_{0}+\Delta t}\langle E_{k}\left(n,t_{0}\right)\rangle dt;$$

where *k* denotes the range, *n* is the sequence number of EEG signal, $t_{0}^{\prime }$ is the discrete time for the function (5) and its step is the same as Δt. Thus, the two-dimensional function (5) allows performing a spatio-temporal analysis of oscillatory activity based on the estimates of the energy values in different frequency ranges. It also allows to conduct spatio-temporal analysis of EEG recordings from the estimates of the interrelation between the CWT energy values for different types of oscillation activity. In Figure 1c we show the results of the energy estimated in the band [8;12] Hz by each EEG channel with a time step Δt.

In order to estimate the total energy of the wavelet transformation per alpha wave range, we used $\langle E^{EEG}\rangle$, computed as:

(6) $$\langle E\rangle =\sum_{k=1}^{5}\sum_{n=1}^{N}\langle E_{k}\left(n,t_{0}^{\prime }\right)\rangle .$$

where *N* represents the number of EEG channels.

Mean, median, and standard deviation were used in descriptive statistics of the data. The Mann-Whitney U test for independent samples was used for the comparison of quantitative data [47]. The results with a *p*-value ≤0.001 were considered to show statistical significance. Statistical analyses were performed in the SPSS version 22.0 program for Windows (IBM, Armonk, NY, USA).

## 3. Results

**Figure 2:**
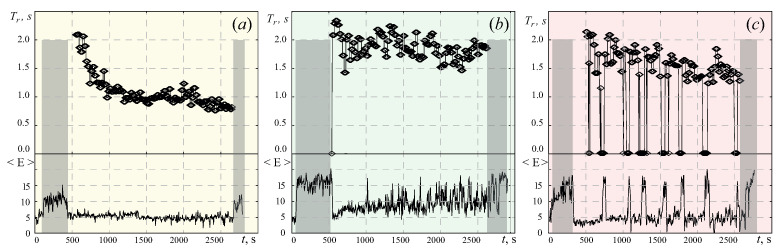
(**a**) The top panel: test subject response delays decreased with experimental time, over subsequent trials to the sound stimulus $T_{r}\left(t\right)$ on experimental time *t*; the bottom panel: the dependence of wavelet energy $\langle E(t)\rangle$ in the frequency interval of [8;12] Hz. These dependences were calculated for test subject #7 from Group I (control group with a healthy sleep); (**b**,**c**) represent similar dependences of $T_{r}\left(t\right)$ and $\langle E(t)\rangle$ for test subject #11 from experimental Group II (with primary insomnia) and for test subject #3 from Group III (with hypersomnia), respectively. Light gray rectangles indicate CY and RCY stages (passive wakefulness of test subjects with closed eyes–initial and repeated).

Response time, $T_{r}$, as a function of experimental time *t* were calculated for each subject. A typical pattern of such dependences is shown on the top panel in Figure 2. The bottom row in Figure 2 demonstrates the corresponding dependences of the total energy $\langle E\rangle$ for the entire frequency range $\Delta f_{\alpha }$. Figure 2a–c demonstrate the characteristics for a participant of the group with a healthy sleep (Group I), insomnia (Group II), and daytime somnolence (Group III), correspondingly.

Passive wakefulness stages with closed eyes, CY and RCY, highlighted in gray in Figure 2, demonstrate the maximum values of $\langle E(t)\rangle$ for test subjects in all three groups. However, during the active stage A, alpha rhythm demonstrates different scenarios of its dynamics. In Group I (the control encompassing subjects with healthy sleep), we observe subject-to-subject variability in dependence of response time $T_{r}$. Among the subjects of this group, $T_{r}$ varies 0.75–3.21 s with the $mean(T_{r})\approx 1.25$ and the $median(T_{r})\approx 1.21$. The overall dynamics of alpha rhythm energy $\langle E(t)\rangle$ is characterized by the wide range and variability during the active stage A. We can see some increase in the amplitude of the alpha rhythm at the end of this stage in the control group (see Figure 2a).

For patients with primary insomnia (Group II), we observe a minimum level of alpha rhythm energy during the active stage A, and fluctuations in the amplitude of $\langle E(t)\rangle$ are under 30–40% of the average (see Figure 2b). At the same time, test subjects from this group successfully maintain the state of wakefulness and a quick response to sound signals. The response time $T_{r}$ after the short adaptation period decreases to a minimum, and then there is virtually no increase. In this group, the response time $T_{r}$ is within 0.71–2.8 s range with the mean and the median of response time: $mean(T_{r})\approx 1.02$; $median(T_{r})\approx 0.99$. Thus, the Group II, shows an illustrative example of adaptation to the process of periodic responses to external stimuli, leading to a significant decrease in response time at the end of an active stage A.

As for the Group III, the dependences of $T_{R}\left(t\right)$ and $\langle E(t)\rangle$ for patients with poor sleep hygiene practices and daytime hypersomnia are characterized by the most complex dynamics during the active stage A. Test subjects in this group reveal the periods without responses to sound stimuli. Precise time intervals, at which the subject’s responses are absent, are shown in the upper diagram in Figure 2c, using zero values of the $T_{r}\left(t\right)$ dependence. The duration of similar states without responses to the sounds for different subjects in the group varies from 38–437 s. The response time $T_{r}$ in Group III ranges between 0.8 and 7.2 s, and its median and mean are quite similar: $mean(T_{r})\approx 1.34$; $median(T_{r})\approx 1.35$. On average, the total duration of stages without responses for subjects in this group is 397.98±213.30 s.

It is worth noting that the subjects of the Groups I and II do not exhibit the long periods of “missed” sound stimuli (the largest amount of the latter is 4). During such periods without the subject’s response, we observe a powerful increase in alpha activity, which, most likely, is associated with involuntary eye closure and the state of a micro-sleep [48]. We also observe that, at some stages, the energy level $\langle E(t)\rangle$ can exceed the value recorded during the passive wakefulness stages with closed eyes—CY and RCY.

Further on, we consider how the activity changes at each band Δf=1 Hz within the alpha rhythm. For each group of subjects, we compare the change in energy $E_{RCY/CY}$ at the stages of passive wakefulness with closed eyes, CY and RCY, with the change in energy $E_{A}$ during an active stage A. Hence, we estimate the value of $E_{RCY/CY}$ and $E_{A}$ according to the following expressions:

(7) $$E_{RCY/CY}=\frac{\sum_{RCY}\langle E_{\Delta f}\rangle }{\sum_{CY}\langle E_{\Delta f}\rangle },$$

and also the value $E_{A}$:

(8) $$E_{A}=\frac{\sum_{\left(A_{end-300}\right)}^{A_{end}}\langle E_{\Delta f}\rangle }{\sum_{A_{0}}^{\left(A_{0+300}\right)}\langle E_{\Delta f}\rangle },$$

where RCY and CY are the registration durations for stages RCY and CY, respectively; $A_{end}$ is the time of registration of the last sound stimulus during the stage A; $\left(A_{end-300}\right)$ is a moment of time 300 s before the last sound stimulus during the stage A; $\left(A_{0+300}\right)$ is a moment of time 300 s after the beginning of the stage A; $A_{0}$ is the registration time of the first sound stimulus during stage A.

**Figure 3:**
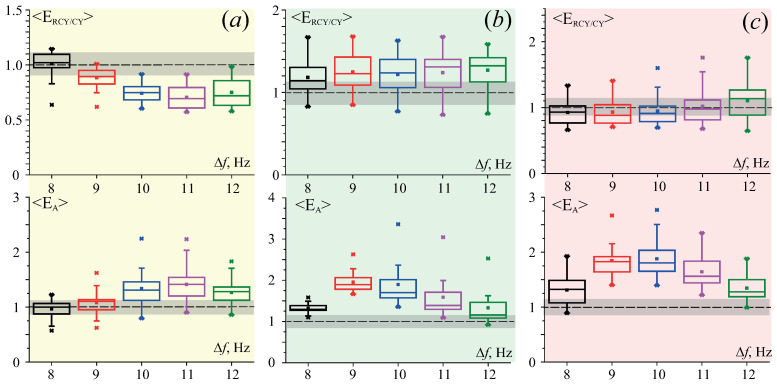
(**a**–**c**) The diagrams of energy ratio $E_{RCY/CY}$ (7) (top panel) and $E_{A}$ (8) (bottom panel) by frequency interval $\Delta f\in \Delta f_{\alpha }$ for I, II, and III participant’s groups, respectively. The diagrams depict the following statistical characteristics of energy ratios: the first and the third quartiles (25–75%, inside the box); the median and mean (dash line and point inside the box, accordingly); 1.5 interquartile range (shown by whiskers); and outliers represented by asterisks. Light gray rectangles delineate the ranges of values representing the ratios close to one.

Figure 3 illustrates the statistical assessment results of the distribution of these quantities in three groups of subjects. Figure 3a presents the data for Group I: it shows an increase in the alpha rhythm activity at all frequency intervals Δf during the repeated passive wakefulness stage RCY in comparison with the initial passive wakefulness stage CY. At the same time, the dynamics of alpha activity at the active stage A are less homogeneous. Frequency interval Δf=[9;10] shows the maximum increase in alpha rhythm energy in the EEG at the end of an active stage A; and with increase in frequency, the alpha rhythm level declines.

For Group II, the energy variables, $E_{RCY/CY}$ and $E_{A}$, remain constant in the frequency interval Δf=[8;9], and as the frequency increases, these dependences are in antiphase, i.e., the energy $E_{RCY}$ of alpha rhythm during second passive stage undergoes reduction, compared with the energy $E_{CY}$ of first passive stage, and the energy $E_{A}$ of the active stage A increases (see Figure 3b, top and bottom panels, accordingly). For test subjects in Group III, the ratio $E_{RCY/CY}$ does not change, whereas $E_{A}$ rises dramatically to the maximum value at 10 Hz (see Figure 3c).

Table 1 presents the estimation results of the Wilcoxon test used for comparing independent data on the ratio of wavelet energies by channels. p<0.001 is considered to confirm statistical significance. We used the stricter threshold of statistical significance to reduce the number of possible false detected differences [49,50]. All *p*-values below this threshold are marked in bold. Hence, for faster oscillatory activity in alpha rhythm (frequency range f∈[9.5;12.5] Hz) at passive wakefulness stages (CY and RCY), significant differences are observed for analyzed EEG results of all study groups.

At an active stage A, significant differences between the experimental groups shift to a lower frequency zone of the alpha rhythm (frequency range f∈[7.5;10.5] Hz). In the process of a cognitive activity, it is possible to distinguish between the subjects with a healthy sleep (Group I), insomnia (Group II), and daytime somnolence (Group III). However, the dynamics of alpha rhythm in patients with insomnia (Group II) and daytime sleepiness (Group III) during the cognitive processes does not show significant differences at any of investigated frequencies (f∈[7.5;12.5]).

## 4. Discussion

Modern pathophysiological concepts of chronic insomnia link its development and transition into a chronic form with the central nervous system hyperarousal. Nonetheless, the manifestation mechanisms of such hyperarousal during the daytime hours, activation mechanisms in the evening, as well as its compensatory mechanisms under insomnia, still remain not fully known.

In this regard, studies of daytime activity in patients with insomnia attract substantial attention. The common feature of such projects involves a better understanding of the brain activity in subjects with insomnia, combined with their psychological traits. For example, a recent research by Losert et al. (2020) reported a daytime increase in the vigilance stability measured by the vigilance algorithm Leipzig (VIGALL) algorithm in patients with insomnia [51]. In earlier studies, it was quite successfully demonstrated that patients with insomnia exhibited a reduction in the power of a low-frequency activity, along with a rise in the power of a high-frequency activity [10,12,14]. At the same time, Kwan et al. showed that the indicated EEG changes in patients with insomnia were not specific and coincided with those in patients with depression [23]. In this article, we additionally studied structural changes of the alpha rhythm in the EEG of the study subjects during daytime psychophysiological tests.

Young people of similar ages with a normal BMI without cognitive and emotional impairments were selected to participate in our experimental work, distributed among the groups according to the nature of their sleep disorders, if any. Quantitative analysis of alpha activity during daytime studies showed significant differences in brain activity of patients with various types of insomnia, both among themselves and from the control group subjects. For the stage of the cognitive experiment, we discovered a steady increase in the alpha rhythm level in subjects with a healthy sleep during the stages of a passive rest. This increase was associated with a prolonged, gradual relaxation in a darkened room, which correlated with some previously published results [4,39]. Patients of Group III did not display significant differences from those with healthy sleep in the frequency interval of [7.5;9.5] Hz. At the same time, in Group II subjects, the alpha rhythm dynamics was fundamentally different: it decreased at the end of the experiment, i.e., the ratio $E_{RCY/CY}\geq 1$ was true solely for patients with primary insomnia. In our opinion, this finding unequivocally demonstrates the difference between the psychophysiological insomnia, caused by the peculiarities of the central nervous system functioning, and acquired sleep disorders. Besides, it is interesting to note that the increase in alpha activity in patients with poor sleep hygiene is virtually unnoticeable. In other words, somnolence seems to lead to an initially high level of alpha activity, which stops increasing further on.

However, brain activity during the stage A in the course of the subject’s reaction to monotonous sound signals shows less pronounced differences. In particular, patients with normal sleep cannot be reliably categorized by alpha activity scores versus those with poor sleep hygiene. Subjects of Group III demonstrated the maximum increase in the alpha rhythm with prolonged exposure to the cognitive function of attention, while patients of Group II even demonstrated a decrease in the intensity of the alpha rhythm for low-frequency components. It can be assumed that, in this case, different compensatory mechanisms for prolonged cognitive exposure in patients with sleep hygiene disorders vs. those with primary insomnia are clearly noticeable. In the former, long-term monotonous cognitive activity (e.g., responding to the sound stimuli) causes intolerable drowsiness and overall relaxation. According to [52] a similar effect could be caused by the paradoxical inhibition of the arousal in the nervous system during monotonous external stimulation. Simultaneously, the overall decrease in the concentration ability in such patients, manifested in augmented response time to stimuli, is consistent with the study by E. Tonnoli [53].

In patients with primary insomnia, activation and control of the cognitive function of attention appears just as effective (maybe, even more effective) as in people with healthy sleep. Thus, this characteristic state, generally known as alertness, automatically includes the states that occur in the course of falling asleep, which can be called anxious expectation. This condition increases tension, alertness and, as a consequence, the functional activity of the brain, leading to a decrease in the levels of such objective experimental criteria as reaction time and alpha rhythm.

Moreover, when analyzing the oscillatory structure of the alpha rhythm, it should be noted that significant differences among three groups of subjects during passive rest (CY and RCY stages) are observed in high-frequency alpha wave bands of [9.5;12.5] Hz. For patients with acquired sleep disorders and subjects with primary insomnia, when comparing passive wakefulness stages, significant differences in the alpha rhythm parameters are observed for the entire frequency range. During the active stage of our experimental work, significant changes in the alpha rhythm unfold in the low frequency range of [7.5;10.5] Hz, while poor sleep hygiene in Group III, relative to the control group, does not lead to changes in psychophysiological characteristics. It can be assumed, following the logic of some studies [18,23,51], that for subjects with primary insomnia, attempting to relax at passive wakefulness stages, we observe pronounced processes of increasing beta activity, which also leads to a rise in the power of the alpha rhythm at high frequencies, and upon activation of the cognitive function of attention, generates the processes of destroying low-frequency activity.

The limitation of the presented study involves the homogeneity of the studied subjects in terms of their young age and male gender. However, this was an unavoidable step towards an initial clear detection of the changing alpha activity characteristics. The further study should consider age and gender characteristics, and also analyze the stability of identified traits in the inevitably emerging comorbid conditions of the patients. In addition to these major limitations of participants population, local limitations of the study design also exist. They are associated with the difficulty of objective control of the subjects immediately before the experimental work. In this study design, we have not performed polysomnography of the subject’s night-time sleep immediately before the day of the experiment. Additionally, the time spent awake prior testing could influence the results. At the same time, polysomnography and direct control of the time from awakening to experimental work are possible only if the participants stay in a clinical setting for 24 h, which is quite difficult to implement.

In our concluding remarks, we would like to emphasize that our results do not contradict earlier studies and support the hypothesis that insomnia is not just a sleep disorder, neither is it solely a nocturnal disorder of the central nervous system. Rather, it is a systemic condition, involving altered functioning of the central nervous system, and, in particular, the human brain. For subjects in this state, it is possible to objectively demonstrate an increase in the reaction speed to sound stimuli, an overall decrease in the power of the alpha rhythm and, at the same time, an increase in the frequency of oscillations within a EEG alpha-rhythm. Besides, the psychophysiological characteristics of patients with sleep hygiene disorders without primary insomnia demonstrate exceptional dynamics: a decrease in the reaction rate, the maximum increase in the alpha rhythm during cognitive exposure, and the absence of the alpha rhythm changes at the resting stages. Hence, we propose that the developed technique may be applied to clarify the impact level of poor sleep hygiene on insomnia in different groups of patients.

Today, digital and computerized methods of the cognitive behavioral therapy for insomnia (dCBT-I) have become increasingly important [54,55,56], caused by the expensive and difficult to obtain quality individual treatment, based on classic CBT-I. Such computer systems have great potential as a basis for the development of systems with biofeedback, including EEG neurofeedback [57]. In this case, the proposed detection of the power level of EEG oscillation on different frequency bands within the alpha-rhythm can be used as an objective control of the patient’s state with sleep disorder complaints. In addition, it is possible that the relaxation training of the subject using the control of alpha-activity on the EEG to achieve the indicators characteristic of a normally sleeping person can also become an base on development of dCBT-I in the direction of the brain-computer interfaces (BCI). Today, the possibility of a quick study of the BCI-prospects can be implemented, for example, within the CRED checklist [58]. Additionally, we hypothesize that regular training alpha activity of the brain towards alpha rhythm in subjects with healthy sleep may be useful in general therapy for insomnia.

## 5. Conclusions

The results of the current study supported the notion of a 24-h hyperarousal in primary insomnia. Tracking the alpha rhythm activity by employing wavelet methods could be a pragmatic option for diagnostic purposes, as well as for BCI development in experimental studies on neuropsychological therapy of primary insomnia, using feedback, within the framework of cognitive attention tests.

## Figures and Tables

**Table 1:** Analysis of the energy ratio ERCY/CY and EA for all groups in different frequency bands. ***m*** is a median; **Δ** is the standard deviation for each frequency interval for the corresponding group; **P1** is the *p* value of the Group I (control) compared with the Group II (patients with primary insomnia); **P2** is the *p* value of the Group II compared with the Group III (patients with daytime somnolence); **P3** is the *p* value of the Group III compared with the Group I (control). All values of p<0.001 characterize statistically significant differences and are highlighted in bold. Frequency bands are indicated as follows: $\Delta f_{1}$ = 7.5–8.5 Hz, $\Delta f_{2}$ = 8.5–9.5 Hz, $\Delta f_{3}$ = 9.5–10.5 Hz, $\Delta f_{4}$ = 10.5–11.5 Hz, $\Delta f_{5}$ = 11.5–12.5 Hz, $\Delta f_{\alpha }$ = 7.5–12.5 Hz.

Frequency Band	Group I		Group II		Groupe III		*P*1	*P*2	*P*3
	m	δ	m	δ	m	δ			
$E_{\mathrm{RCY}/\mathrm{CY}}$									
$\Delta f_{1}$	1.14	0.211	1.02	0.105	0.929	0.168	0.001	**<0.001**	0.038
$\Delta f_{2}$	1.22	0.22	0.895	0.086	0.882	0.201	**<0.001**	**<0.001**	0.799
$\Delta f_{3}$	1.238	0.225	0.748	0.08	0.909	0.23	**<0.001**	**<0.001**	**<0.001**
$\Delta f_{4}$	1.31	0.235	0.694	0.1	0.978	0.246	**<0.001**	**<0.001**	**<0.001**
$\Delta f_{5}$	1.324	0.218	0.72	0.128	1.135	0.26	**<0.001**	**<0.001**	**<0.001**
$\Delta f_{\alpha }$	1.274	0.211	0.837	0.084	0.953	0.207	**<0.001**	**<0.001**	**<0.001**
$E_{A}$									
$\Delta f_{1}$	1.295	0.103	1.000	0.149	1.322	0.249	**<0.001**	0.531	**<0.001**
$\Delta f_{2}$	1.895	0.257	1.104	0.199	1.827	0.269	**<0.001**	0.068	**<0.001**
$\Delta f_{3}$	1.699	0.521	1.308	0.295	1.803	0.319	0.001	0.891	**<0.001**
$\Delta f_{4}$	1.389	0.509	1.413	0.278	1.562	0.300	0.378	0.17	0.011
$\Delta f_{5}$	1.159	0.422	1.28	0.207	1.278	0.237	0.953	0.493	0.256
$\Delta f_{\alpha }$	7.42	1.635	6.15	1.1	7.864	1.177	0.001	0.891	**<0.001**

## Data Availability

The data that support the findings of this study are available from the corresponding author upon reasonable request.

## Abbreviations

The following abbreviations are used in this manuscript:

A-stage	Active experimental stage: wakefulness with periodic sound stimuli
CY	First experimental stage of passive wakefulness with closed eyes
RCY	Second experimental stage of passive wakefulness with closed eyes
CWT	Continuous wavelet transform
